# LncRNA SNHG25 Predicts Poor Prognosis and Promotes Progression in Osteosarcoma *via* the miR-497-5p/SOX4 Axis

**DOI:** 10.2174/1386207326666230602122618

**Published:** 2024-03-07

**Authors:** Ningjun Wan, Qiang Liu, Jiandang Shi, Siliang Wang

**Affiliations:** 1 Ningxia Medical University, Yinchuan, Ningxia, China;; 2 Department of Orthopedics, People’s Hospital of Ningxia Hui Autonomous Region, Yinchuan, Ningxia, China;; 3 Department of Orthopedics, General Hospital of Ningxia Medical University, Yinchuan, Ningxia, China

**Keywords:** LncRNA SNHG25, osteosarcoma, miR-497-5p, tumour progression, SOX4, biomarker

## Abstract

**Background:**

Osteosarcoma is a disease that primarily affects adolescents with skeletal immaturity. LncRNAs are abnormally expressed and correlated with osteosarcoma patients' prognosis. We identified aberrant expression of LncRNA SNHG25 (small nucleolar RNA host gene 25) in osteosarcoma and analyzed the molecular mechanisms by which it regulates osteosarcoma progression.

**Methods:**

The expression levels of SNHG25 in tumour specimens and cells were measured by RT-qPCR. Loss-of-function assays were conducted to investigate the functional role of SNHG25 *in vitro* and *in vivo*. Bioinformatic predictions, dual-luciferase reporter assays, and western blotting were performed to explore the possible underlying mechanisms.

**Results:**

SNHG25 was highly expressed in osteosarcoma cells and tissues. The Kaplan–Meier curve showed that the survival rate of patients with high SNHG25 expression was significantly lower than those with low SNHG25 expression. Functional studies have indicated that inhibition of SNHG25 suppresses cell proliferation, migration, and invasion, while promoting apoptosis. SNHG25 knockdown suppresses osteosarcoma tumour growth *in vivo*. SNHG25 functions as a sponge for miR-497-5p in osteosarcoma cells. The level of SNHG25 was negatively correlated with that of miR-497-5p. The proliferation, invasion, and migration of osteosarcoma cells were restored by transfection of the miR-497-5p inhibitor in the SNHG25 knockdown group.

**Conclusion:**

SNHG25 was determined to function as an oncogene by promoting osteosarcoma cell proliferation, invasion, and migration through the miR-497-5p/SOX4 axis. Upregulation of SNHG25 expression indicated poor prognosis in patients with osteosarcoma, which showed that SNHG25 may serve as a potential therapeutic target and prognostic biomarker in osteosarcoma.

## 1 INTRODUCTION

Osteosarcoma is the primary malignant bone tumour affecting children and young adolescents, and it occurs most frequently in the distal femur and proximal tibia [1]. In recent years, the 5-year event-free survival rate of patients with osteosarcoma has increased from less than 20% to approximately 60% owing to neoadjuvant chemotherapy [2, 3]. However, few evidence-based improvements have been introduced to improve the survival of patients [2-5]. There is an urgent need to elucidate the molecular mechanisms underlying osteosarcoma development to identify new therapeutic targets in order to improve patient prognosis.

LncRNAs are a novel class of RNA molecules defined as transcripts containing > 200 nucleotides transcribed by RNA polymerase II [6]. Harrison *et al*. [7] found the proportion of tumour-related LncRNA genes to be approximately twice that of tumour-related protein-coding genes. LncRNAs are abnormally expressed in gastric, lung, and pancreatic cancers, hepatocellular carcinoma, osteosarcoma, and other malignant tumours, and mediate tumour progression by regulating cell growth, apoptosis, and metastasis [8-10]. The expression of members of the small nucleolar RNA host gene family is correlated with Enneking staging, tumour size, histological tumour grade, and clinical prognosis in osteosarcoma patients. For example, SNHG12 is significantly upregulated in osteosarcoma tissues and cell lines, and osteosarcoma patients with high levels of SNHG12 tend to have a poor prognosis [11]. The expression of SNHG5 is elevated in patients with osteosarcoma, which correlates with poor clinical outcomes and prognosis [12]. However, the role of SNHG25 in osteosarcoma remains unclear.

SNHG25 is a newly discovered LncRNA involved in tumour progression located on chromosome 17q23.3. The LncRNA SNHG25 was first identified to be highly expressed in epithelial ovarian cancer, which promotes progression by regulating cartilage oligomeric matrix protein [13]. In addition, SNHG25 is involved in the progression of malignant endometrial cancer [14]. LncRNAs in the cytoplasm compete with endogenous RNAs (ceRNAs) to modulate the development of human cancers [15, 16]. The LncRNA TUG1 promotes osteosarcoma cell proliferation and invasion by upregulating ezrin expression as a ceRNA of miR-377-3p [17]. MiRNAs are small, noncoding, regulatory RNA molecules, ranging between 18 and 25 nucleotides in length [18]. MiRNAs bind to sequences with partial complementarity on target RNA transcripts, called miRNA recognition elements (MREs), usually resulting in the repression of target gene expression [19, 20]. MiRNA-497-5p suppressed tumour cell growth in osteosarcoma by targeting ADP-ribosylation factor-like protein 2 [21]. Using bioinformatic tools, miR-497-5p was found to bind to SNHG25 and target SOX4 (SRY-box transcription factor 4).

In view of the fact that SNHG25 plays an essential role in promoting cancer in several malignant tumours, we aimed to elucidate our hypothesis that SNHG25 functions as an oncogene by promoting osteosarcoma cell proliferation, invasion, and migration through the miR-497-5p/SOX4 axis, thus showing potential as a biomarker and therapeutic target for osteosarcoma.

## 2 MATERIALS AND METHODS

### 2.1 Clinical Samples and Cell Culture

This study was approved by the ethics committee of the General Hospital of Ningxia Medical University (No. KYLL-2021-562), and a written informed consent form was signed by all patients. Ten osteosarcoma samples and matched adjacent normal tissues were collected from August 2021 to August 2022, and postoperative pathology indicated osteosarcoma. The specimens were frozen and stored at -80°C until use.

Osteosarcoma cell lines (U-2OS, MG-63, and Saos-2) and a normal osteoblast cell line (hFOB 1.19) were purchased from Procell Life Science & Technology (Wuhan, Hubei, China), and verified using STR genotyping. U-2OS and Saos-2 cells were cultured in McCoy's 5A medium supplemented with 10% FBS (fetal bovine serum) and 1% penicillin and streptomycin. MG-63 cells were grown in minimum essential medium supplemented with 10% FBS and 1% penicillin and streptomycin. hFOB 1.19 cells were cultured in F12 medium and Dulbecco’s modified Eagle medium supplemented with 0.3 mg/ml G418, 10% FBS, and 1% penicillin and streptomycin. hFOB 1.19 cells were maintained in an incubator at 34°C with 5% CO_2_, and all other cells were maintained at 37°C with 5% CO_2_.

### 2.2 Bioinformatics Analyses and Survival Analysis

We investigated the most differentially expressed genes between osteosarcoma tissues and normal adjacent tissues using the GEO database (https://www.ncbi.nlm.nih.gov/geo/query/acc.cgi?acc=GSE126209). We then generated a heatmap based on the differences in gene expression. We downloaded gene expression data and survival time, survival status, and follow-up time of 88 osteosarcoma patients from the Therapeutically Applicable Research to Generate Effective Treatments (TARGET) database (https://xenabrowser.net/datapages/). The median expression of the SNHG25 and SOX4 was calculated and divided into high and low-expression groups. Forty-four patients were included in each of the two groups. The Kaplan‒Meier plotter was used to compare the overall survival rate between the high-expression and low-expression groups by GraphPad Prism 8. The Log-rank test was used to analyze whether there was a difference in the overall survival rate between the high-expression and low-expression groups at ten years of follow-up.

### 2.3 Total RNA Extraction and RT‒qPCR

Total RNA was extracted from the cells and tissues using TRIzol reagent (Invitrogen, California, USA). After measuring the concentration of total RNA, 1μg of total RNA was reverse-transcribed into cDNA using 5X PrimeScript RT Master Mix (Takara, Dalian, China) for SNHG25 and SOX4 expression analysis. GAPDH was used as the reference control for SNHG25 and SOX4 expression. RT-qPCR was performed using TB Green^®^ Premix Ex Taq™ (Takara, Dalian, China) and Applied Biosystems QuantStudio 5 system (Thermo Fisher Scientific, Massachusetts, USA) with the following thermal cycling program: 95°C for 30 s, followed by 40 cycles at 95°C for 5 s and 60°C for 31 s. Poly(A) tails were added to the 3' ends of the miRNAs using a miRcute Plus miRNA first-strand cDNA kit (TIANGEN Biotech Co., Ltd., Beijing, China). Reverse transcription was performed using reverse transcription primers to synthesize first-strand cDNA corresponding to miRNA. RT-qPCR was performed using a miRcute Plus miRNA qPCR kit (TIANGEN Biotech Co., Ltd., Beijing, China) and an Applied Biosystems QuantStudio 5 system with the following thermal cycling program: 95°C for 15 min, followed by 40 cycles at 94°C for 20 s and 60°C for 34 s. U6 served as the reference gene for miR-497-5p. The data were analyzed using the 2^-△△Ct^ method. All primers used are listed in Table **1**.

### 2.4 Cell Infection and Transfection

To achieve a stable knockdown of SNHG25 expression, two short hairpin RNAs (shRNAs) targeting SNHG25 were synthesized by GenePharma (Shanghai, China) (Table **2**). MG-63 and U-2OS cells were seeded in six-well plates. When the cells were 40% confluent, 5μg/ml of polybrene and the appropriate amount of lentivirus were added to the culture medium. We added puromycin to select stably transduced cells when GFP expression was observed after 72h of infection. SOX4 was inserted into the pcDNA3.1 vector to construct pcDNA3.1-SOX4. According to standard protocols, all plasmids were transfected into osteosarcoma cell lines (MG-63 and U-2OS) using Lipo2000 (Invitrogen, California, USA).

### 2.5 CCK-8 Assay and Flow Cytometric Analysis

The CCK-8 assay was used to evaluate cell proliferation. Stably transfected cells were prepared in a suspension at a concentration of 4×10^4^ cells/ml. Transfected osteosarcoma cells (100 µl) were inoculated into each well of 96-well plates and cultured for 24, 48, 72, or 96 h. CCK-8 reagent (10 µl) was added to each well (KeyGEN BioTECH, Nanjing, China) and incubated in a 5% CO_2_ incubator at 37°C for 3 h. A microplate reader was used to measure the absorbance of each well at 450 nm wavelength.

Apoptosis was evaluated using flow cytometry. Stably transfected cells were prepared in a suspension at a concentration of 1-10×10^5^ cells/ml. A binding buffer was then used to suspend the cells. Annexin V-FITC (5 µl) and propidium iodide (10 µl; Multi-Science, Hangzhou, Zhejiang, China) were added to each tube. The cells were then incubated for 5 min at 37°C in the dark.

### 2.6 Transwell and Wound Healing Assay

A wound-healing assay was used to evaluate cell migration. Cells were inoculated into 6-well plates. When the cells were 90% confluent, a 200-µl pipette tip was used to make vertical scratches along a horizontal marker line. Serum-free culture medium was added after washing thrice with PBS. Micrographs of the scratches were acquired at 0 and 24 h, and ImageJ software was used to assess the wound area.

Costar transwell inserts were used for transwell assays to determine cell invasion ability (Yunshan Biotech, Sichuan, China). The Matrigel matrix was diluted with serum-free medium and added to each upper chamber. Cells were prepared as a suspension with a concentration of 5 × 10^5^ cells/ml, and 100 µl of the osteosarcoma cells with the serum-free medium were inoculated into each upper chamber and cultured at 37°C for 24 h. Transwell inserts were removed and washed with PBS thrice. The non-invaded cells and Matrigel matrix were removed from the upper chamber membranes by wiping them with cotton swabs. Crystal violet (0.1%) was used for staining after fixation with 4% glutaraldehyde. Micrographs of the cells were acquired, and ImageJ software was used to count the cells.

### 2.7 Subcellular Fractionation Location

MG-63 and U-2OS cells were collected, and total cytoplasmic and nuclear RNA was extracted using the instructions of a cytoplasmic and nuclear RNA extraction kit (Beibei Biotechnology, Zhengzhou, China). 8 µl cytoplasmic and nuclear total RNA was extracted and configured with a 10µl reaction system to reverse-transcribe into cDNA, respectively. Finally, cDNAs from cytoplasmic and nuclear fractions were determined by qPCR assays as described above. U6 was used as the control of nuclear transcript, and GAPDH was used as the control of cytoplasmic transcript.

### 2.8 Luciferase Reporter Assay

We predicted the target sites for binding SNHG25, SOX4, and miR-497-5p using the StarBase algorithm (http://starbase.sysu.edu.cn/), and further verified these binding sites using a luciferase reporter assay. All the constructed plasmids were fabricated and verified by sequencing (Wuhan GeneCreate Biological Engineer, China). According to the kit protocol, luciferase activity was measured using a Dual-Luciferase Reporter Gene Assay Kit (Beyotime Biotechnology, Shanghai, China). The pmirGLO-SNHG25-WT or pmirGLO-SNHG25-MUT plasmids were co-transfected with miR-NC or miR-497-5p mimic into 293T cells using Lipo2000 (Invitrogen, California, USA) (Table **2**). Next, 293T cells were co-transfected with the WT or MUT SOX4 plasmid and the miR-497-5p mimic or NC using the Lipo2000 transfection system protocol. Firefly luciferase activity was normalized to Renilla luciferase activity 48 h after transfection by using a chemiluminescence image analyzer (Supplementary Material).

### 2.9 Protein Extraction and Western Blotting

The total protein was extracted using RIPA buffer (Beyotime Biotechnology, Shanghai, China). After the protein concentrations were measured using a BCA kit (KeyGEN BioTECH, Nanjing, China), 30 µg of protein was separated using 8–12% sodium dodecyl sulfate-polyacrylamide gel electrophoresis and transferred to polyvinylidene difluoride membranes (Merck, Millipore, Germany). The membranes were blocked with 5% skim milk powder at room temperature for 2 h and subsequently incubated at 4°C for 12 h with primary antibodies specific for the following proteins: SOX4 (1:200, Santa Cruz Biotechnology, California, USA), PCNA (1:3000; Proteintech Group, Wuhan, China), and GAPDH (1:3000; Affinity Biosciences, Changzhou, China). After incubation with an IRDye 800CW secondary antibody for 40 min, protein bands were visualized using the Odyssey CLx Image Studio software (LI-COR, Shanghai, China).

### 2.10 Tumour Growth Assay in Nude Mice

Twelve female BALB/c nude mice (5 weeks of age) were purchased from Beijing Vital River Laboratory Animal Technology Co. Ltd. (Beijing, China) and randomly divided into two groups. MG-63 cells were then infected with sh-NC or sh-SNHG25-2. Thereafter, 150 µl of infected cells (5 × 10^7^ cells/mouse) were injected subcutaneously into the right axilla of the mice. Tumours were allowed to grow for 22 days. Tumour volumes and body weights of the mice were recorded every three days. Tumour volume was calculated using the following formula: volume (mm^3^) = length ×width^2^ × 0.5.

### 2.11 Statistical Analyses

All data analyses were performed using SPSS 23.0 and GraphPad Prism 8, and the data have been presented as mean ± SD. A paired t-test was conducted to compare the expression between the tumour and normal tissues. The Student’s t-test was used to compare the normal and control groups' differences. Comparisons of continuous variables among multiple groups were made using ANOVA (Bonferroni). Overall survival analysis was performed by the Kaplan–Meier method. The correlation between SNHG25 and miR-497-5p, miR-497-5p and SOX4, and SNHG25 and SOX4 gene expression in osteosarcoma tissues was analyzed by the Pearson method. Differences were considered statistically significant at *P* < 0.05.

## 3 RESULTS

### 3.1 SNHG25 Expression is Upregulated in Osteosarcoma Tissues and Cells and it Predicts a Poor Prognosis in Osteosarcoma Patients

**Fig. (1):**
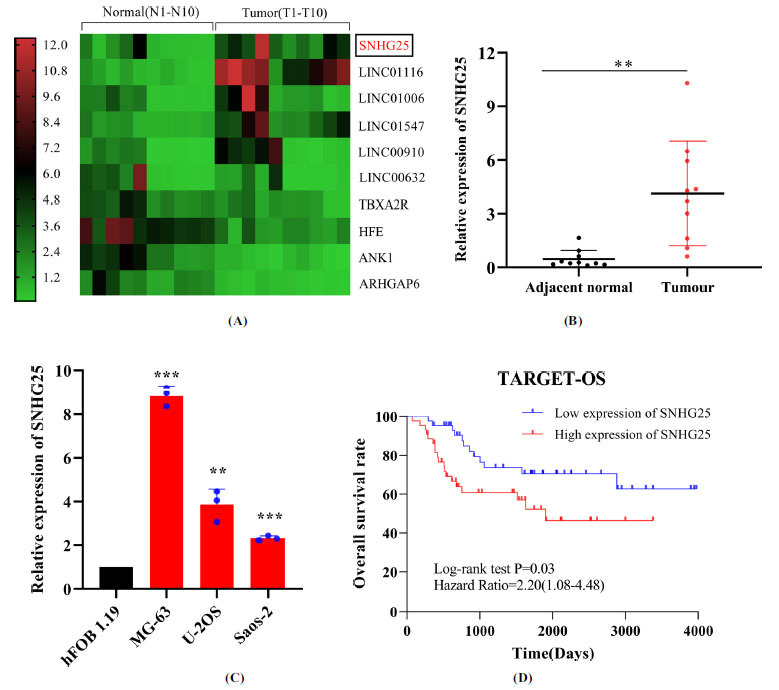
SNHG25 is upregulated in osteosarcoma tissues and cells, predicting an unfavorable prognosis in osteosarcoma patients. (**A**) Heatmap of differentially expressed RNAs in osteosarcoma samples in the GEO database (GSE126209). (**B**) RT‒qPCR was performed to determine the SNHG25 level in ten paired osteosarcoma tumour tissues and adjacent normal tissues. (**C**) SNHG25 expression was shown in osteosarcoma cells (MG-63, U-2OS, and Saos-2) compared to normal osteoblasts (hFOB 1.19). (**D**) Kaplan–Meier analysis showed the survival rates of patients with high expression (n=44) and low expression (n=44) of SNHG25 (*P*=0.03). The data are shown as mean ± SD. **P < 0.01, ***P < 0.001.

The heatmap indicated the SNHG25 expression level to be dramatically elevated in osteosarcoma tissues compared to normal adjacent tissues (n=10) (Fig. **1A**). We collected ten pairs of osteosarcoma tissues and paracancerous normal tissues for evaluation of SNHG25 expression using RT–qPCR. SNHG25 was found to be highly expressed in osteosarcoma tissues compared to normal adjacent tissues (Fig. **1B**). In addition, SNHG25 expression was higher in osteosarcoma cells (MG-63, U-2OS, and Saos-2) than in normal osteoblasts (Fig. **1C**). Kaplan–Meier survival curve showed the survival rate of patients with high SNHG25 expression (n=44) to be significantly lower than that of patients with low SNHG25 expression (n=44) in the TARGET database (Fig. **1D**). The 10-year overall survival rate of osteosarcoma patients in the low-expression SNHG25 group was 72.7%, and that in the high-expression group was 56.8%, as assessed by Log-rank test (*P*=0.03). More importantly, the Log-rank analysis confirmed increased SNHG25 expression to be an unfavorable prognostic factor for osteosarcoma patients (HR=2.20, 95% CI: 1.08–4.48, *P*=0.03).

### 3.2 SNHG25 Knockdown Inhibits Osteosarcoma Cells' Proliferation, Migration, and Invasion, and it Promotes Apoptosis *in vitro*

**Fig. (2):**
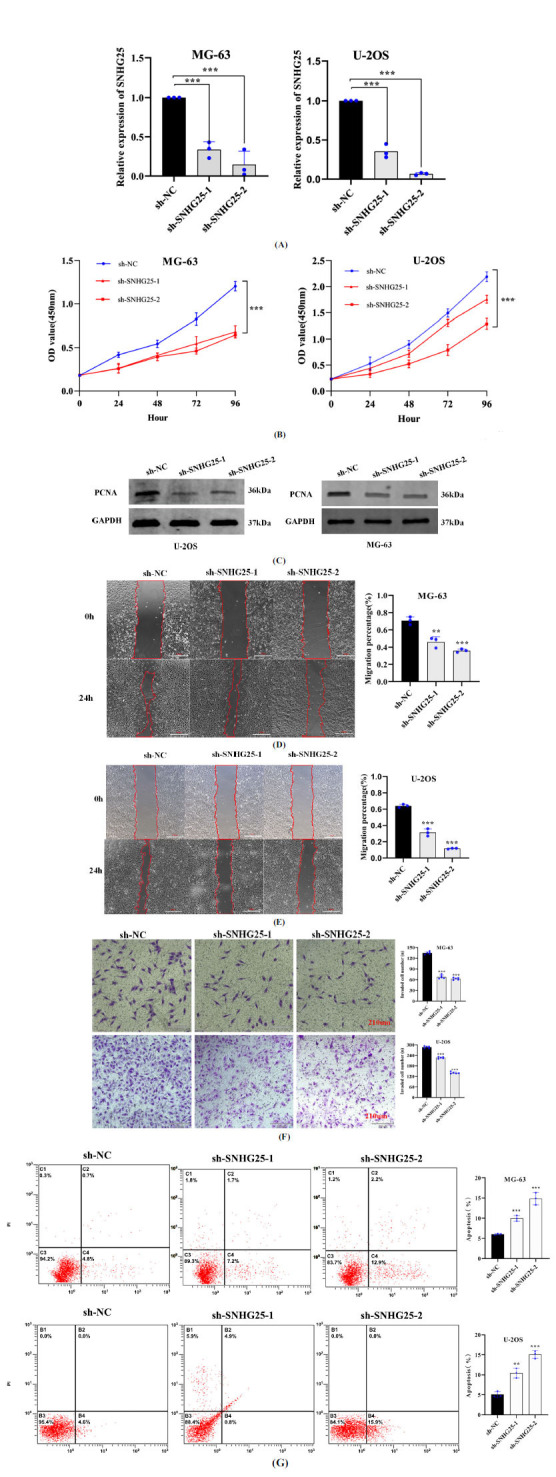
Knockdown of SNHG25 inhibited osteosarcoma cells' proliferation, invasion, and migration, and promoted their apoptosis. (**A**) MG-63 and U-2OS cells were infected with lentivirus carrying shRNA specifically targeting SNHG25, and RT‒qPCR was used to analyse the shRNA knockdown efficiency. (**B**) CCK-8 assays were performed to evaluate the proliferation. (**C**) The PCNA protein expression was detected by western blotting. (**D-F**) Wound healing and transwell assays were performed to evaluate migration and invasion. (**G**) Apoptosis was analysed by flow cytometry. The data are presented as mean ± SD of three independent experiments (scale bar, 560 µm or 210µm). *P < 0.05, **P < 0.01, ***P < 0.001.

We infected osteosarcoma cells with lentiviruses loaded with sh-SNHG25-1 and sh-SNHG25-2. The RT-qPCR results showed the expression of SNHG25 to be significantly inhibited in the sh-SNHG25-1 and sh-SNHG25-2 group (Fig. **2A**). CCK-8 assay showed the proliferation ability of osteosarcoma cells to be dramatically reduced in the knockdown group after 48 h of culture (Fig. **2B**). Western blotting showed PCNA protein expression to be significantly downregulated in the knockdown group, which indicated the cell proliferation as decreased (Fig. **2C**). Wound healing and transwell assays indicated that SNHG25 knockdown inhibited osteosarcoma cell migration and invasion (Fig. **2D-2F**). Flow cytometry results showed the apoptosis rate of osteosarcoma cells to be markedly increased after SNHG25 knockdown (Fig. **2G**).

### 3.3 SNHG25 Functions as a Sponge for Mir-497-5p in Osteosarcoma Cells

**Fig. (3):**
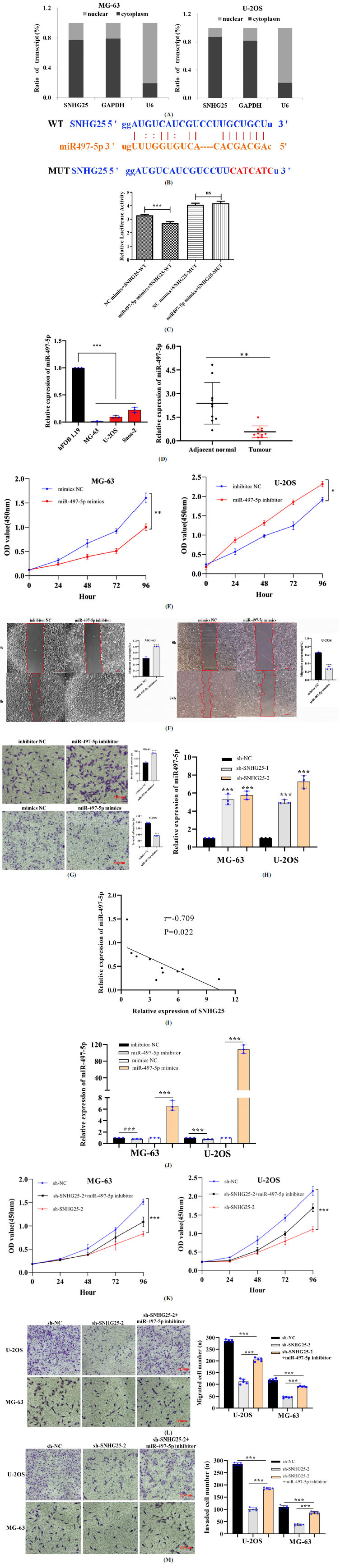
SNHG25 functions as a sponge for miR-497-5p in osteosarcoma cells. (**A**) Subcellular location detection for SNHG25 in MG-63 and U-2OS cells. (**B**) The predicted site for binding between miR-497-5p and SNHG25. (**C**) Effects of the miR-497-5p mimic luciferase activity driven by the WT and MUT SNHG25 constructs. (**D**) MiR-497-5p expression was downregulated in osteosarcoma tissue and cell lines. (**E**) CCK-8 assays were performed to evaluate the proliferation after transfection with the miR-497-5p mimic or miR-497-5p inhibitor. (**F** and **G**) The miR-497-5p mimic and miR-497-5p inhibitor affect the migration and invasion. (**H**) MiR-497-5p levels in osteosarcoma cells were assessed after SNHG25 knockdown. (**I**) Correlation between SNHG25 expression and miR-497-5p expression in osteosarcoma tissues. (**J**) MiR-497-5p levels in osteosarcoma cells were assessed after transfection of the miR-497-5p mimic or miR-497-5p inhibitor. (**K**) Effects of sh-SNHG25-2 and the miR-497-5p inhibitor on the proliferation. (**L** and **M**) Effects of sh-SNHG25-2 and the miR-497-5p inhibitor on migration and invasion (scale bar, 560 µm or 210 µm). *P<0.05, **P<0.01, ***P<0.001.

Subcellular fractionation assays showed SNHG25 to be mainly expressed in the cytoplasm of osteosarcoma cells (Fig. **3A**). Therefore, SNHG25 might exert its oncogenic functions by acting as a miRNA sponge. We predicted the target sites for binding between SNHG25 and miR-497-5p by using the StarBase algorithm and verified by luciferase reporter assay (Figs. **3B** and **3C**). The results proved SNHG25 to be capable of interacting with miR-497-5p. Subsequently, we found miR-497-5p expression to be downregulated in osteosarcoma tissue and cell lines (Fig. **3D**). Cell functional assays showed that miR-497-5p inhibited osteosarcoma cell proliferation, invasion, and migration (Fig. **3E-3G**). Moreover, miR-497-5p expression in the osteosarcoma cell lines was upregulated after SNHG25 knockdown (Fig. **3H**). The expression levels of SNHG25 and miR-497-5p were found to be negatively correlated (Fig. **3I**). MiR-497-5p expression was found to be upregulated or downregulated after transfection with the miR-497-5p mimic or miR-497-5p inhibitor, respectively (Fig. **3J**). The proliferation, invasion, and migration of osteosarcoma cells were restored by transfection with the miR-497-5p inhibitor in the SNHG25 knockdown group (Fig. **3K-3M**). Therefore, the above data prove miR-497-5p to be a direct target of SNHG25 in osteosarcoma cells.

### 3.4 Mir-497-5p Targets SOX4 in Osteosarcoma Cells

**Fig. (4):**
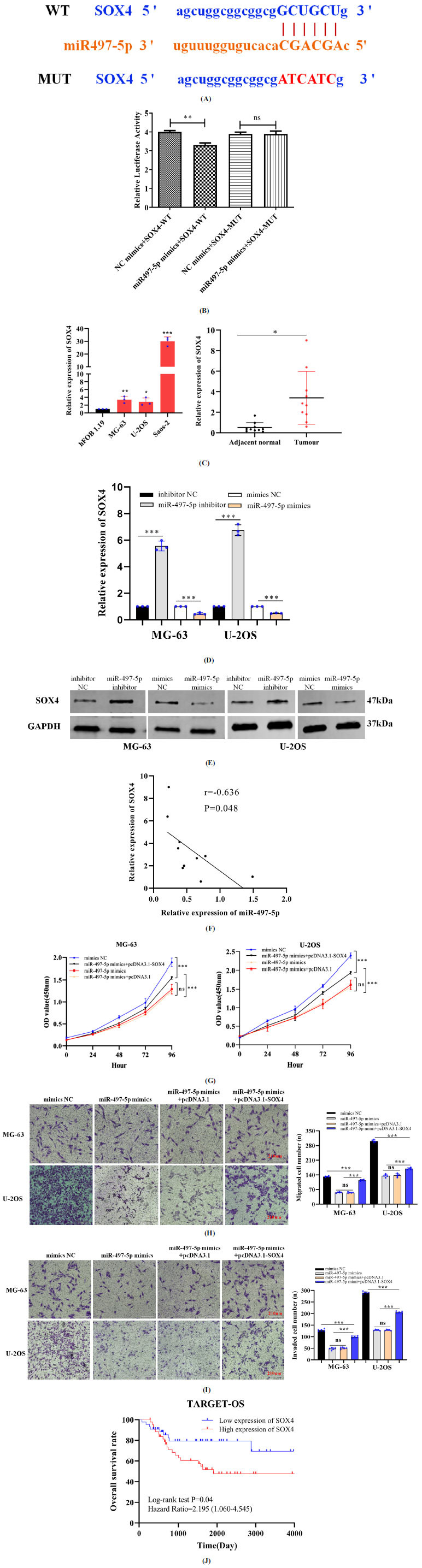
miR-497-5p targets SOX4 in osteosarcoma cells. (**A**) The predicted site for binding between miR-497-5p and SOX4. (**B**) Effects of the miR-497-5p mimic luciferase activity driven by the WT and MUT SOX4 constructs. (**C**) SOX4 expression in osteosarcoma cells and tissues. (**D** and **E**) SOX4 mRNA and protein expression were detected after transfected with the miR-497-5p inhibitor or miR-497-5p mimics. (**F**) The SNHG25 expression was positively correlated with SOX4 expression in osteosarcoma tissues. (**G**) CCK-8 assays were performed to evaluate the proliferation of MG-63 and U-2OS cells. (**H** and **I**) Effects of the pcDNA3.1-SOX4 on the migration and invasion of MG-63 and U-2OS cells in miR497-5p mimics group. (**J**) Kaplan–Meier analysis showed the survival rates of patients with high expression (n=44) and low expression (n=44) of SOX4 (*P*=0.04) (scale bar, 210 µm). *P<0.05, **P<0.01, ***P<0.001.

We predicted that miR-497-5p targets SOX4 using the StarBase algorithm, which identified a site for binding between miR-497-5p and the 3'-UTR of SOX4 (Fig. **4A**). The results of luciferase activity assays confirmed miR-497-5p to directly target SOX4 (Fig. **4B**). SOX4 mRNA expression was significantly upregulated in osteosarcoma cells and tissues (Fig. **4C**). The effects of miR-497-5p mimic and inhibitor on SOX4 mRNA and protein expression in osteosarcoma cells were evaluated. Compared to the control group, SOX4 mRNA and protein expression were significantly increased in osteosarcoma cells transfected with the miR-497-5p inhibitor (Figs. **4D** and **4E**). In contrast, transfection with miR-497-5p mimic decreased the mRNA and protein expression levels of SOX4 (Figs. **4D** and **4E**). The expression levels of miR-497-5p and SOX4 were negatively correlated (Fig. **4F**). The proliferation, invasion, and migration of osteosarcoma cells were restored by transfection with pcDNA3.1-SOX4 in the miR-497-5p mimics group (Fig. **4G-4I**). The results demonstrated SOX4 to be a direct downstream target of miR-497-5p in osteosarcoma cells. The 10-year overall survival rate of osteosarcoma patients in the low-expression SOX4 group was 79.5%, and that in the high-expression group was 54.5%, as assessed by the Log-rank test (*P*=0.04). The Log-rank analysis confirmed increased SOX4 expression to be an adverse prognostic factor for osteosarcoma patients (HR=2.195, 95% CI: 1.060–4.545, *P* = 0.04) (Fig. **4J**).

### 3.5 SNHG25 Promotes Osteosarcoma Progression through SOX4

**Fig. (5):**
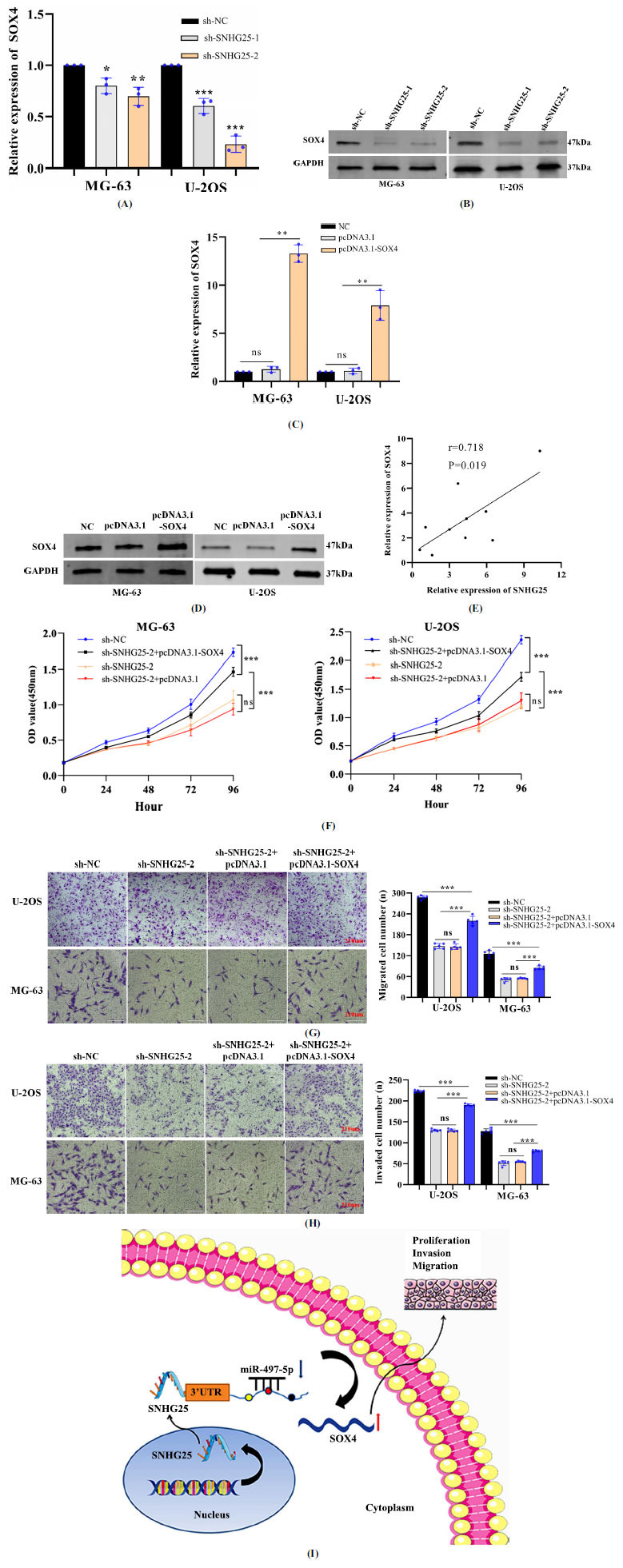
SNHG25 promotes osteosarcoma progression through SOX4. (**A** and **B**) SOX4 mRNA and protein expression after SNHG25 knockdown. (**C** and **D**) SOX4 mRNA and protein expression after SOX4 upregulation. (**E**) Correlation between SNHG25 expression and SOX4 expression in osteosarcoma tissues. (**F-H**) Effects of sh-SNHG25-2 and pcDNA3.1-SOX4 on the proliferation, migration, and invasion of MG-63 and U-2OS cells. (**I**) Summary of the regulation and mechanism of SNHG25 in osteosarcoma (scale bar, 210 µm). *P<0.05, **P<0.01, ***P<0.001.

Next, we sought to determine whether SNHG25 promotes osteosarcoma progression through SOX4. Compared to sh-NC transduction, SNHG25 knockdown significantly downregulated SOX4 expression at both the mRNA and protein levels (Figs. **5A** and **5B**). SOX4 expression in MG-63 and U-2OS cells significantly increased after transfection with pcDNA3.1-SOX4 (Figs. **5C** and **5D**). The expression levels of SNHG25 and SOX4 were found to be positively correlated (Fig. **5E**). Moreover, the proliferation, migration, and invasion of osteosarcoma cell lines were considerably inhibited by SNHG25 silencing, and this inhibition was partially reversed following SOX4 overexpression (Fig. **5F-5H**). These findings suggest that SNHG25 may be oncogenic in osteosarcoma through the miR-497-5p/SOX4 axis (Fig. **5I**).

### 3.6 SNHG25 Knockdown Suppresses the Growth of Osteosarcoma Tumours *in vivo*

**Fig. (6):**
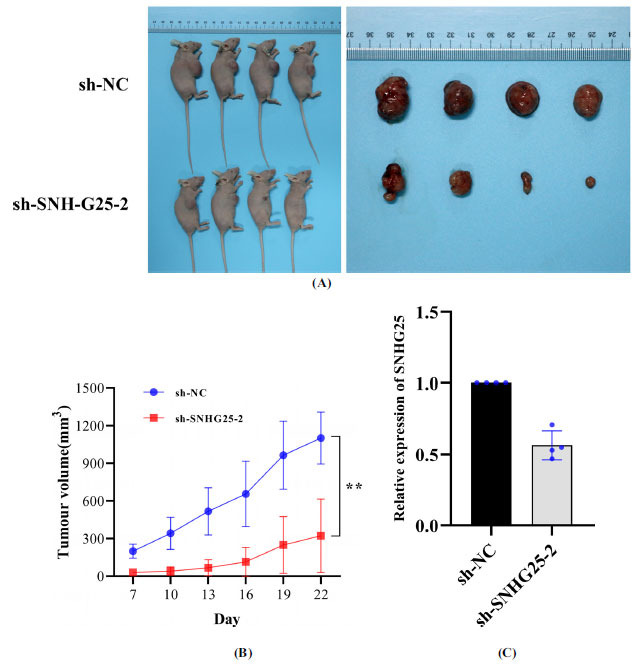
SNHG25 knockdown suppressed the growth of osteosarcoma tumours *in vivo*. (**A**) Effects of SNHG25 knockdown in MG-63 cells on subcutaneous tumour growth. (**B**) Images of whole tumours from nude mice injected with MG-63 cells stably infected with the sh-NC or sh-SNHG25-2 lentiviral vector. (**C**) SNHG25 expression in the sh-NC and sh-SNHG25-2 group. The data are presented as mean ± SD; **P < 0.01.

MG-63 cells were separately infected with sh-NC and sh-SNHG25-2 lentiviruses. Tumours were allowed to grow for 22 days and the mice were sacrificed. In two mice in the sh-NC group and two in the sh-SNHG25-2 group, tumours failed to form 2 weeks after the subcutaneous injection of cells. Tumour growth curves showed that SNHG25-2 depletion markedly reduced tumour volume (Fig. **6A**). Tumour volumes in the sh-NC group were dramatically higher than those in the SNHG25 knockdown group (Fig. **6B**). SNHG25 expression was lower in sh-SNHG25-2 group than the sh-NC group (Fig. **6C**). These data confirm that SNHG25 knockdown suppressed the growth of osteosarcoma tumours *in vivo*.

## 4 DISCUSSION

Osteosarcoma is a deadly disease that primarily affects adolescents with skeletal immaturity [22, 23]. Therefore, there is an urgent need to identify sensitive diagnostic and prognostic biomarkers for osteosarcoma. In recent years, LncRNAs have been recognized as potential novel biomarkers for many tumours. LncRNAs are abnormally expressed in osteosarcoma tissues compared to normal tissues and are involved in osteosarcoma's proliferation, invasion, migration, and apoptosis, which has caused widespread concern [24-26]. Recent studies have shown SNHG1, SNHG5, SNHG16, and SNHG20 to be overexpressed in osteosarcoma tissues and play vital roles in promoting tumour progression [27-30].

In this study, we identified a novel osteosarcoma-related LncRNA, SNHG25, one of the most upregulated LncRNAs in osteosarcoma tissues, by analyzing GEO data. Subsequent RT‒qPCR results showed SNHG25 to be significantly overexpressed in osteosarcoma tissues and cells compared to its normal counterparts. More importantly, SNHG25 knockdown dramatically suppressed the proliferation, migration, and invasion of osteosarcoma cells, and promoted their apoptosis *in vitro*. Moreover, the survival rate of patients in the high-SNHG25-expression group was markedly lower than that of patients in the low-SNHG25-expression group. These results indicate that SNHG25 might serve as an oncogene in osteosarcoma and predict poor prognosis in osteosarcoma patients. However, the underlying mechanism remains unknown.

Previous studies have indicated that LncRNAs can sponge miRNAs, and thus regulate the expression of protein-coding genes, playing a regulatory role in the progression of multiple types of cancers, such as hepatocellular carcinoma, colorectal cancer, and endometrial cancer [31-33]. We identified a binding site between miR-497-5p and SNHG25 using the StarBase algorithm and verified it by luciferase reporter assay. The results have proven SNHG25 to be capable of interacting with miR-497-5p. Cell functional assays have shown miR-497-5p to inhibit osteosarcoma cell proliferation, invasion, and migration. Sun *et al*. [21] found miR-497-5p to be significantly downregulated in osteosarcoma tissues and cells, and to act as a tumour suppressor in osteosarcoma. Ma *et al*. [34] found low expression of miR-497-5p in osteosarcoma tissues and cells. Our cell functional assays demonstrated that miR-497-5p overexpression suppressed osteosarcoma cell proliferation, invasion, and migration. After the knockdown of SNHG25, the expression of miR-497-5p was significantly upregulated. In contrast, transfection with a miR-497-5p inhibitor reversed the effects of SNHG25 knockdown in osteosarcoma cells. Moreover, the rescue assay showed the proliferation, migration, and invasion to be restored in the SNHG25 knockdown group after transfection with the miR-497-5p inhibitor. Therefore, SNHG25 functions as a sponge for miR-497-5p in osteosarcoma cells.

Moreno *et al*. [35] found SOX4 to be overexpressed in 107 (23%) of 462 unique studies in over 20 types of cancer, suggesting SOX4 to be an oncogene. In addition, the recent upregulation of SOX4 in patients with osteosarcoma and its value as a novel prognostic biomarker have been confirmed [36, 37]. In this study, the expression of SOX4 was upregulated in osteosarcoma cell lines and tissues. The survival rate of patients in the high-SOX4-expression group was significantly lower than that of patients in the low-SOX4-expression group. Luciferase activity assays confirmed miR-497-5p to directly target SOX4. Chen *et al*. [38] found SOX4 overexpression to be associated with poor prognosis in osteosarcoma. The knockdown of SOX4 expression in osteosarcoma cell lines decreased cell proliferation, migration, and invasion, and it induced apoptosis. In addition, we found that the inhibition of miR-497-5p expression distinctly increased the expression of SOX4, whereas the upregulation of miR-497-5p demonstrated the opposite effect.

In contrast, SNHG25 silencing downregulated SOX4 expression by binding to miR-497-5p. Moreover, SOX4 overexpression rescued the effects of SNHG25 knockdown on osteosarcoma cell proliferation, migration, and invasion. Finally, we have provided evidence that SNHG25 could influence SOX4 expression as a competing endogenous RNA. However, owing to the limited number of clinical samples, the correlation between SNHG25 expression and Enneking staging, tumour size, histological response to chemotherapy, and lung metastasis of osteosarcoma could not be analyzed in this study. A larger sample size may address the above limitation.

## CONCLUSION

In this study, SNHG25 was found to function as an oncogene by promoting osteosarcoma cell proliferation, invasion, and migration *via* the miR-497-5p/SOX4 axis. Survival analysis showed upregulation of SNHG25 expression to indicate poor prognosis in patients with osteosarcoma. These results indicate that SNHG25 may be a therapeutic target and prognostic biomarker for osteosarcoma.

## Figures and Tables

**Table 1:** The primer sequences used in this study.

**Gene**	**Sequence**
*SNHG25-F*	5'-GCAGGTTCCGGGAGGTCA-3'
*SNHG25-R*	5'-CAAACCACTTTATTGACGGGAA-3'
*miR-497-5p-F*	5'- CCTTCAGCAGCACACTGTGG-3'
*miR-497-5p-R*	5'- CAGTGCAGGGTCCGAGGTAT-3'
*SOX4-F*	5'-CCTTCATGGTGTGGTCGCAGATC-3'
*SOX4-R*	5'-AAGGGATCTTGTCGCTGTCTTTGAG-3'
*GAPDH-F*	5'- GGGCCAAAAGGGTCATCATC-3'
*GAPDH-R*	5'- ATGACCTTGCCCACAGCCTT-3'
*U6-F*	5'-CGCTTCGGCAGCACATATAC-3'
*U6-R*	5'-TTCACGAATTTGCGTGTCAT-3'

**Table 2:** The vector sequences used in this study.

**Vectors**	**Sequence**
sh-NC	5'-TTCTCCGAACGTGTCACGT-3'
sh-SNHG25-1	5'-CCCGTCAATAAAGTGGTTTGA-3'
sh-SNHG25-2	5'-GGATGTCATCGTCCTTGCT-3'
WT-SNHG25	5 ' ggAUGUCAUCGUCCUUGCUGCUu 3 '
MUT-SNHG25	5 ' ggAUGUCAUCGUCCUUCATCATCu 3 '
WT-SOX4	5 ' AGCUGGCGGCGGCGGCUGCUg 3 '
MUT-SOX4	5 ' AGCUGGCGGCGGCGATCATCg 3 '
miR-497-5p mimics	5 'CAGCAGCACACUGUGGUUUGU 3 '
miR-497-5p inhibitor	5 'ACAAACCACAGUGUGCUGCUG 3 '

## Data Availability

The datasets that support the results and findings of this research are available from the corresponding author [J.S.] upon reasonable request.

## LIST OF ABBREVIATIONS

ANOVA: One-Way Analysis Of Variance
ceRNA: Competing Endogenous RNA
GEO: Gene Expression Omnibus
LncRNA: Long Noncoding RNA
MOI: Multiplicity of Infection
MREs: MiRNA Response Elements
RT-qPCR: Quantitative Real-Time Polymerase Chain Reaction
SNHG25: Small Nucleolar RNA Host Gene 25
SOX4: SRY-Box Transcription Factor 4
PCNA: Proliferating Cell Nuclear Antigen
