# Supplementary figures and images for: LncRNA SNHG25 Predicts Poor Prognosis and Promotes Progression in Osteosarcoma via the miR-497-5p/SOX4 Axis
Source: Comb Chem High Throughput Screen. 2024 Mar 7;27(5):725–44. doi: 10.2174/1386207326666230602122618 (PMC11092561; doi:10.2174/1386207326666230602122618)

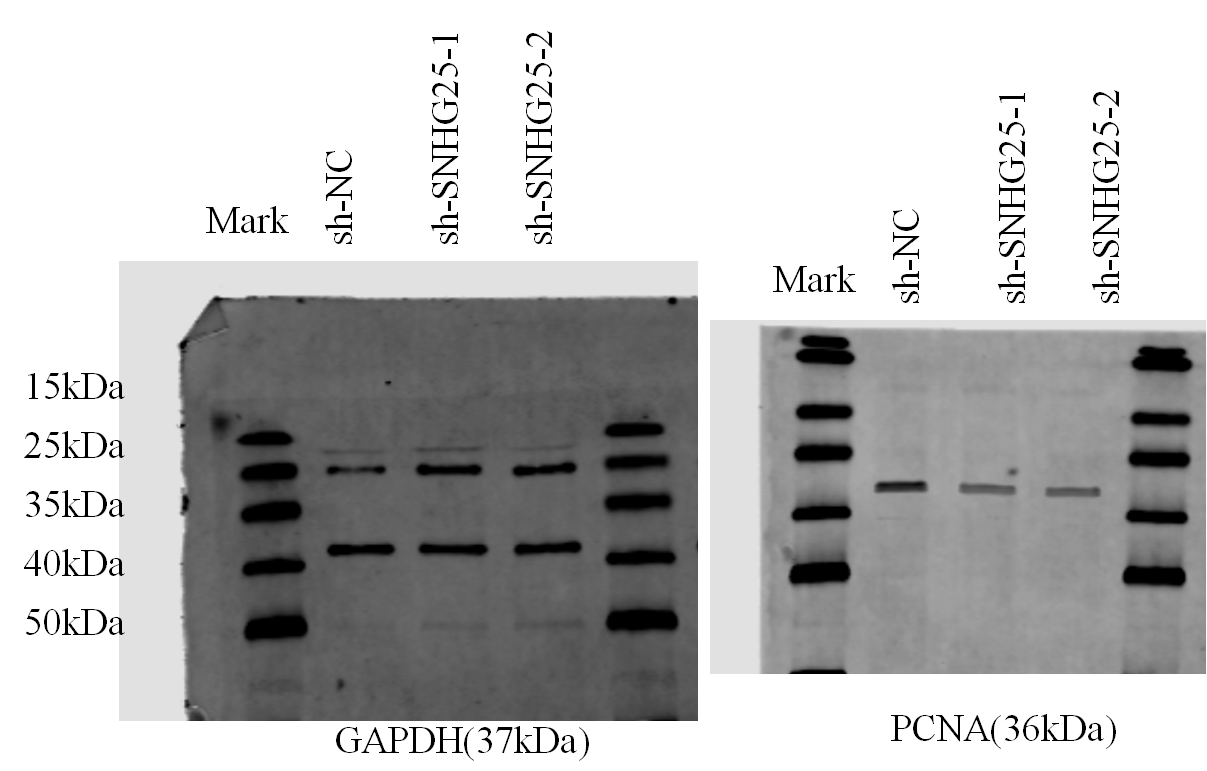

Supplement: Supplementary file 1 — Supplementary material is available on the publisher's website along with the published article. [file CCHTS-27-725_SD1.zip › CCHTS-27-725_SD1/Original image of western blotting/Fig2C-MG-63.tif]

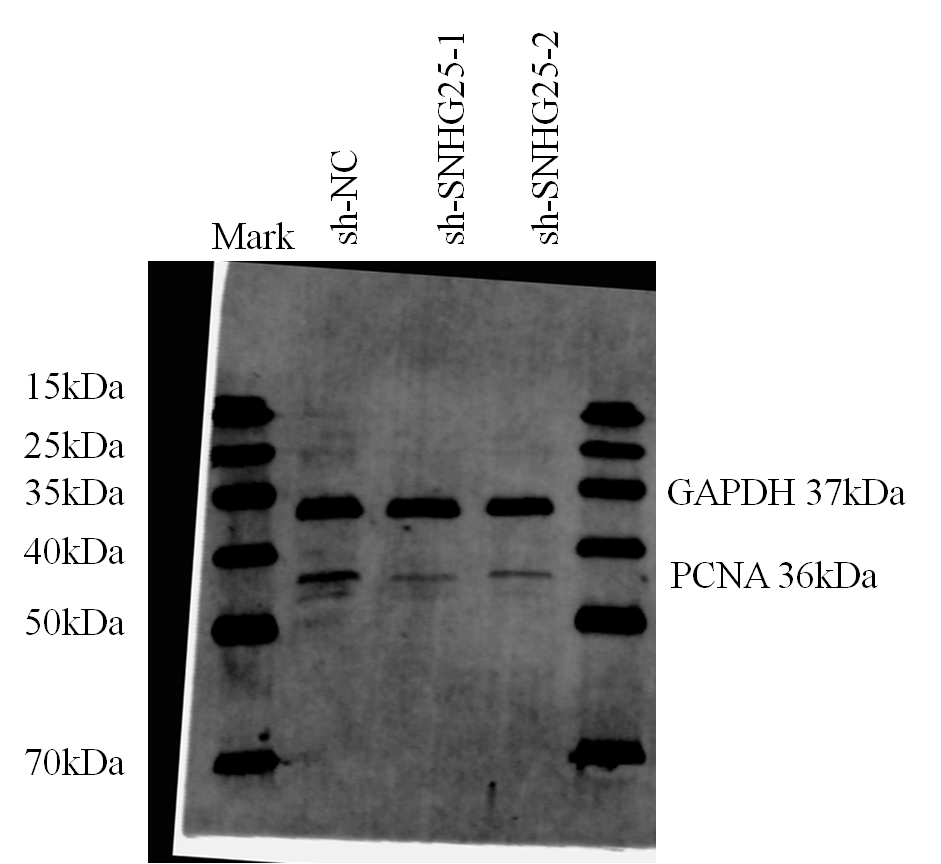

Supplement: Supplementary file 1 — Supplementary material is available on the publisher's website along with the published article. [file CCHTS-27-725_SD1.zip › CCHTS-27-725_SD1/Original image of western blotting/Fig2C-U-2OS.tif]

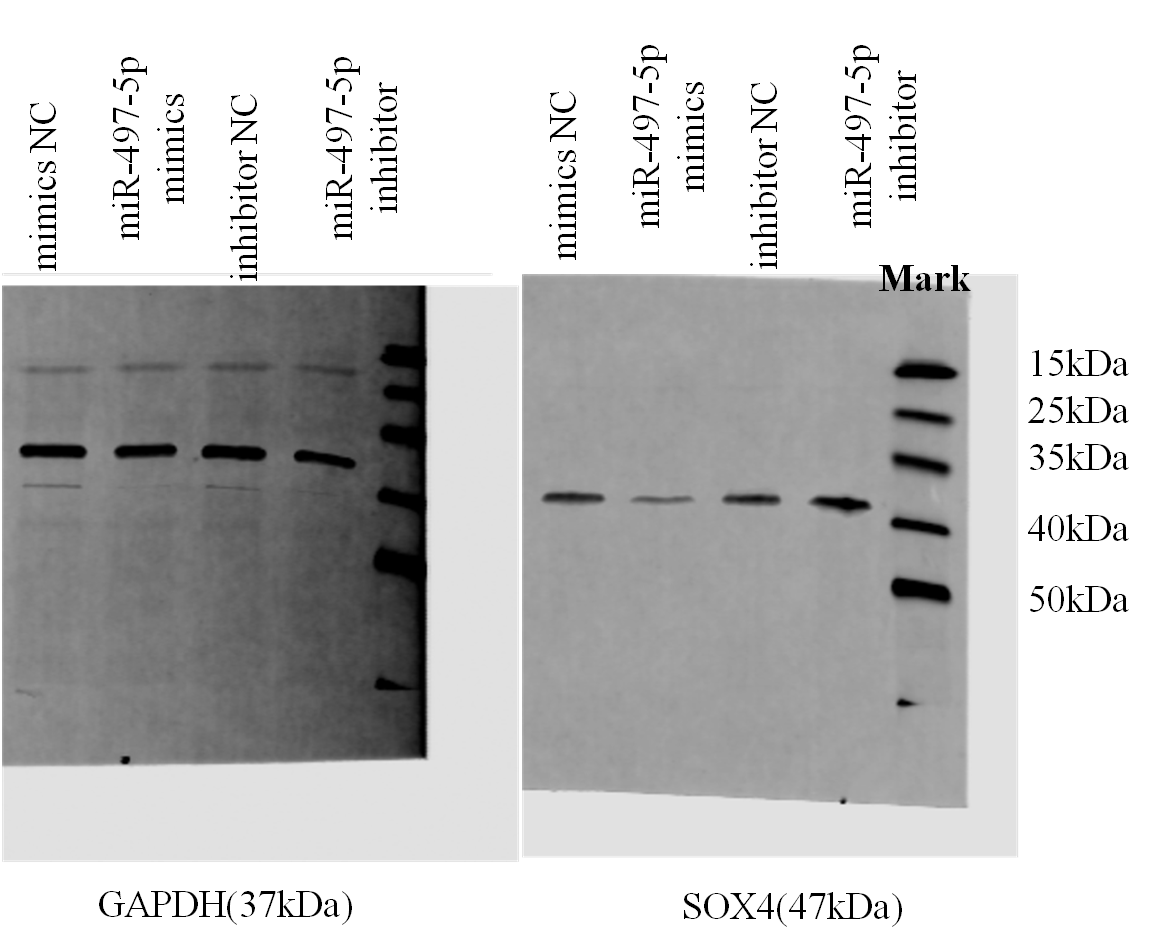

Supplement: Supplementary file 1 — Supplementary material is available on the publisher's website along with the published article. [file CCHTS-27-725_SD1.zip › CCHTS-27-725_SD1/Original image of western blotting/Fig4E-MG-63.tif]

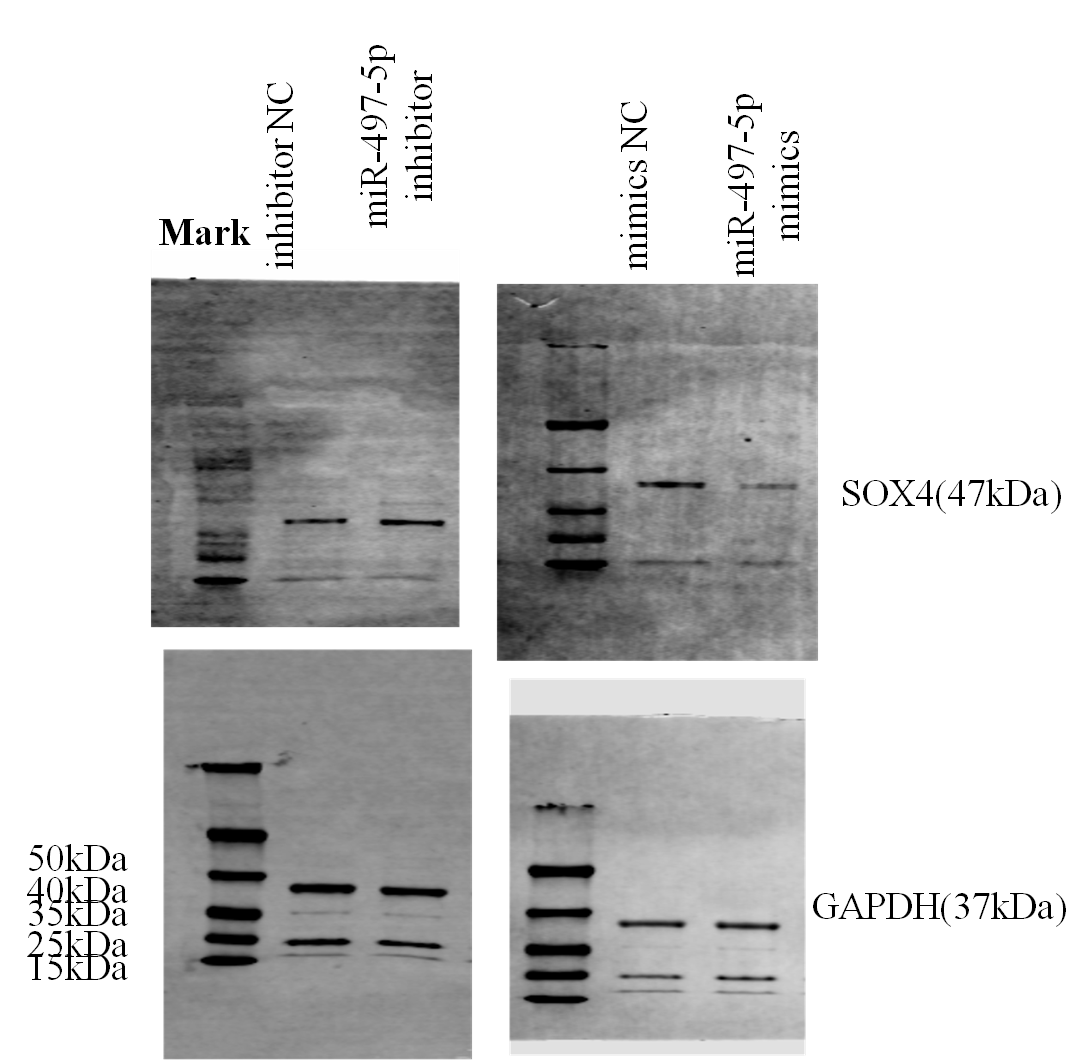

Supplement: Supplementary file 1 — Supplementary material is available on the publisher's website along with the published article. [file CCHTS-27-725_SD1.zip › CCHTS-27-725_SD1/Original image of western blotting/Fig4e-U-2OS.tif]

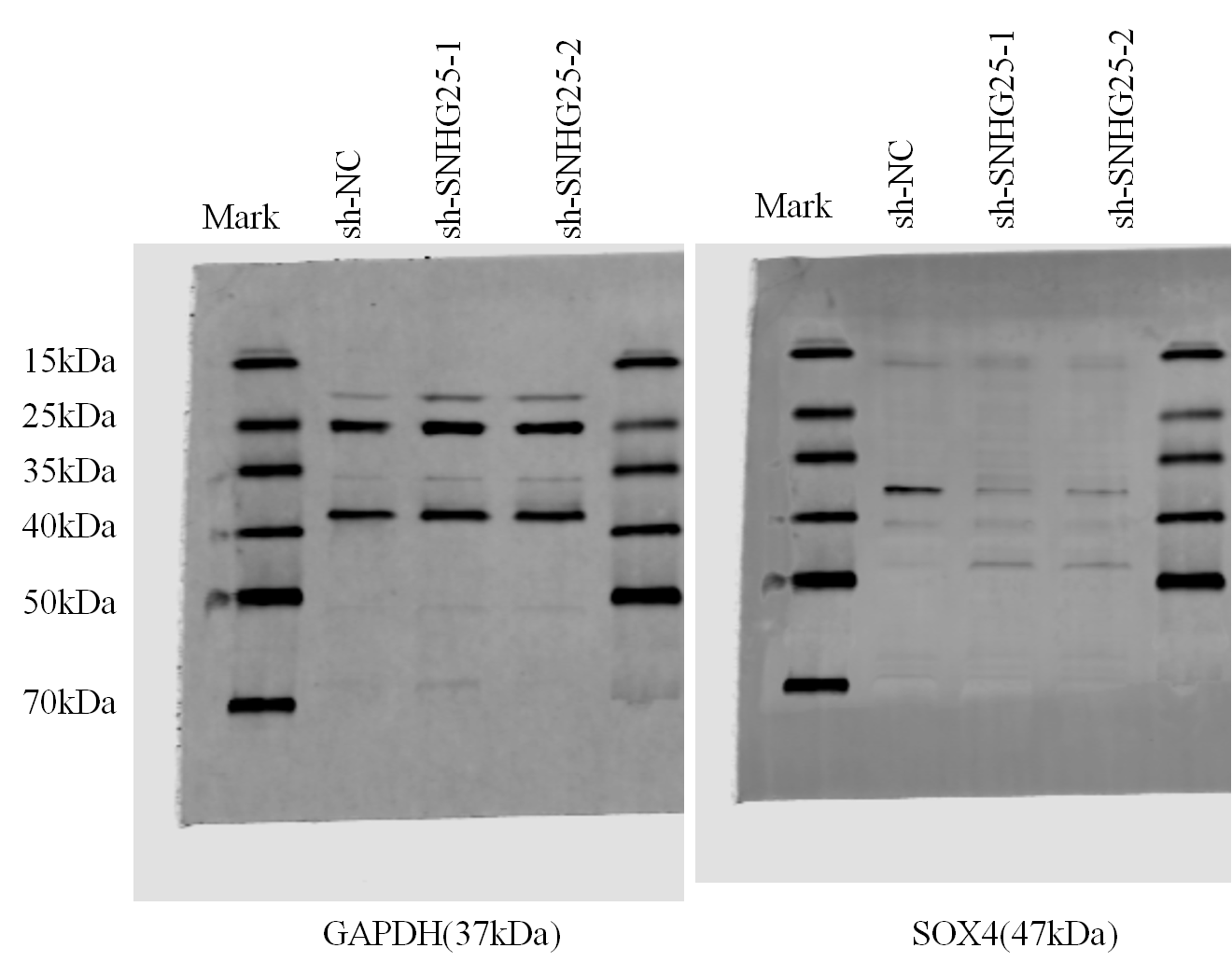

Supplement: Supplementary file 1 — Supplementary material is available on the publisher's website along with the published article. [file CCHTS-27-725_SD1.zip › CCHTS-27-725_SD1/Original image of western blotting/Fig5B-MG-63.tif]

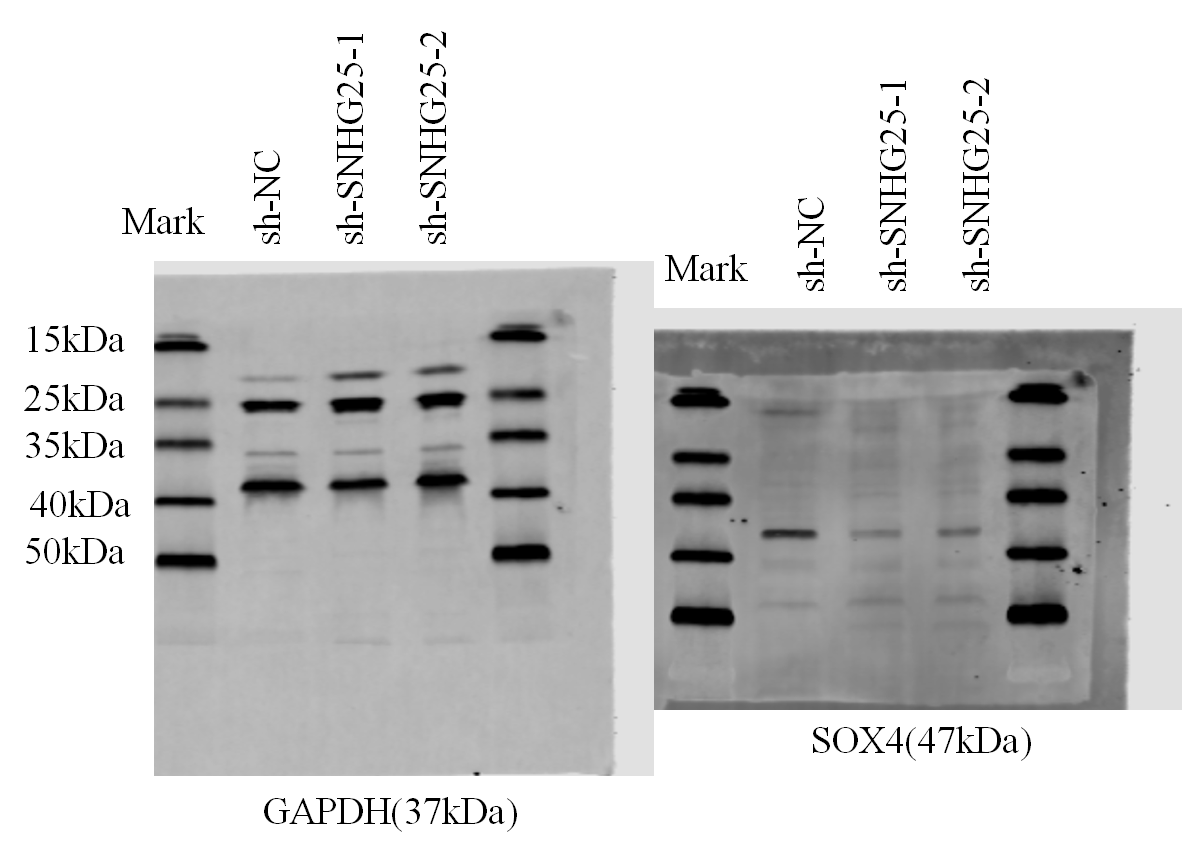

Supplement: Supplementary file 1 — Supplementary material is available on the publisher's website along with the published article. [file CCHTS-27-725_SD1.zip › CCHTS-27-725_SD1/Original image of western blotting/Fig5B-U-2OS.tif]

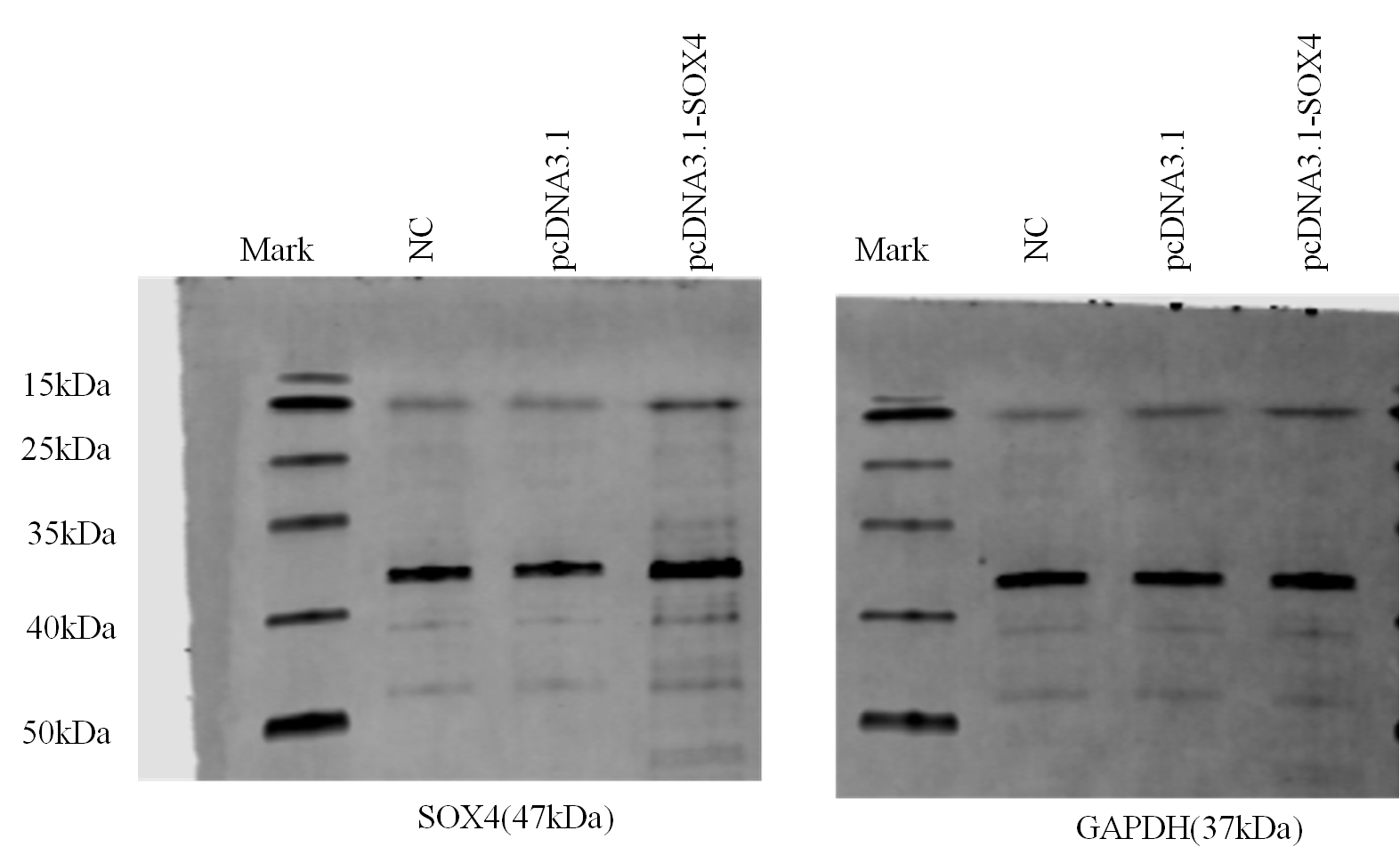

Supplement: Supplementary file 1 — Supplementary material is available on the publisher's website along with the published article. [file CCHTS-27-725_SD1.zip › CCHTS-27-725_SD1/Original image of western blotting/Fig5E-MG-63.tif]

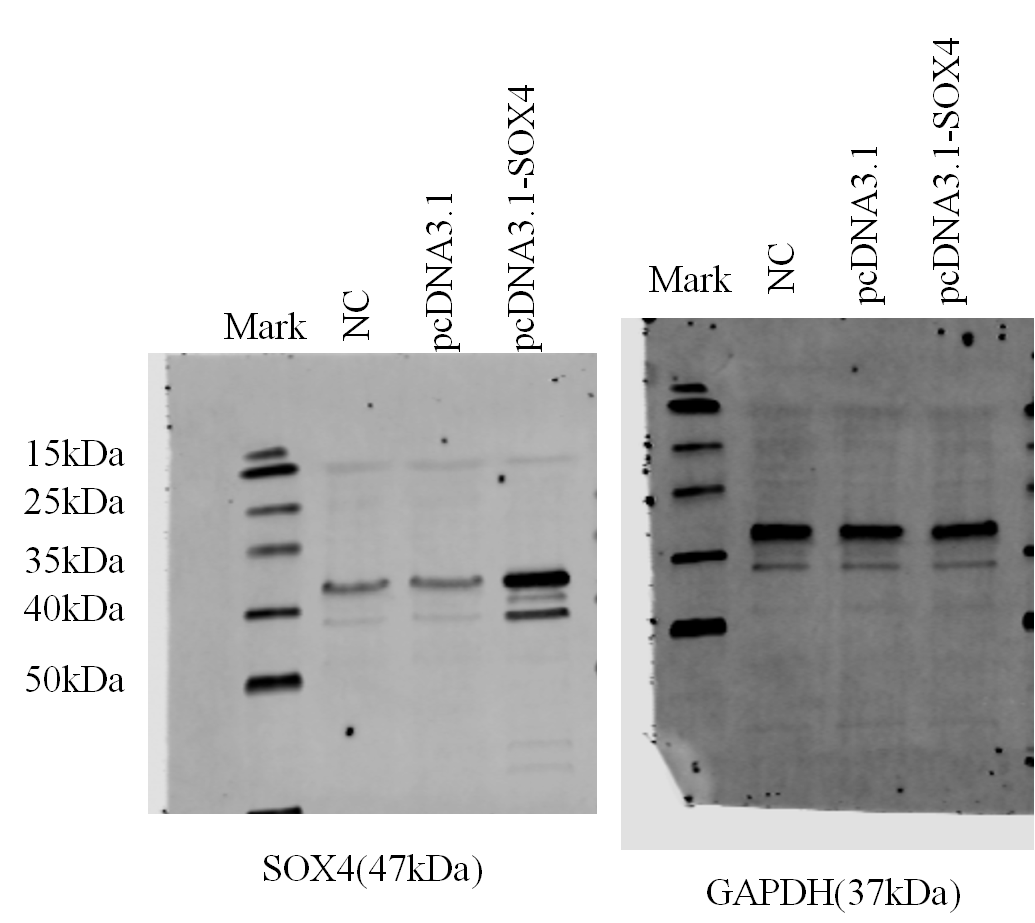

Supplement: Supplementary file 1 — Supplementary material is available on the publisher's website along with the published article. [file CCHTS-27-725_SD1.zip › CCHTS-27-725_SD1/Original image of western blotting/Fig5E-U-2OS.tif]
